# The impact of the COVID-19 pandemic in Malaysia, Indonesia, Thailand and Vietnam: insights from the SEANUTS II study

**DOI:** 10.1017/S1368980024001332

**Published:** 2024-12-20

**Authors:** Jan Geurts, Cécile Singh-Povel, Shoo Thien Lee, Rini Sekartini, Bee Koon Poh, Nipa Rojroongwasinkul, Nga Thuy Tran, Aria Kekalih, Jyh Eiin Wong, Nawarat Vongvimetee, Van Khanh Tran, Ilse Khouw

**Affiliations:** 1 FrieslandCampina, Amersfoort, The Netherlands; 2 Faculty of Health and Life Sciences, Management & Science University, 40100 Shah Alam, Selangor, Malaysia; 3 Department of Child Health, Medical School Universitas Indonesia, Cipto Mangunkusumo General Hospital, Jakarta, Indonesia; 4 Centre for Community Health Studies (ReaCH), Faculty of Health Sciences, Universiti Kebangsaan Malaysia, Kuala Lumpur, Malaysia; 5 Institute of Nutrition, Mahidol University, Nakhon Pathom, Thailand; 6 National Institute of Nutrition, Hanoi, Vietnam; 7 Community Medicine Department, Faculty of Medicine, Universitas Indonesia, Jakarta 10320, Indonesia

**Keywords:** COVID-19, Malnutrition, Health and lifestyle, Children, Survey, South-East Asian Nutrition Surveys II, Lockdown

## Abstract

**Objective:**

To describe the economic, lifestyle and nutritional impact of the COVID-19 pandemic on parents, guardians and children in Malaysia, Indonesia, Thailand and Vietnam.

**Design:**

Data from the SEANUTS II cohort were used. Questionnaires, including a COVID-19 questionnaire, were used to study the impact of the pandemic on parents/guardians and their children with respect to work status, household expenditures and children’s dietary intake and lifestyle behaviours.

**Setting:**

Data were collected in Malaysia, Indonesia, Thailand and Vietnam between May 2019 and April 2021.

**Participants:**

In total, 9203 children, aged 0·5–12·9 years, including their parents/guardians.

**Results:**

Children and their families were significantly affected by the pandemic. Although the impact of lockdown measures on children’s food intake has been relatively mild in all countries, food security was negatively impacted, especially in Indonesia. Surprisingly, in Malaysia, lockdown resulted in overall healthier dietary patterns with more basic food groups and less discretionary foods. Consumption of milk/dairy products, however, decreased. In the other countries, intake of most food groups did not change much during lockdown for households based on self-reporting. Only in rural Thailand, some marginal decreases in food intakes during lockdown persisted after lockdown. Physical activity of children, monthly household income and job security of the parents/guardians were negatively affected in all countries due to the pandemic.

**Conclusion:**

The COVID-19 pandemic has significantly impacted societies in South-East Asia. To counteract negative effects, economic measures should be combined with strategies to promote physical activity and eating nutrient-adequate diets to increase resilience of the population.

The crisis resulting from the coronavirus disease 2019 (COVID-19) pandemic has further increased the prevalence of the double burden of malnutrition in young children in low- and middle-income countries^(1,2)^. Even relatively brief lockdowns, combined with severe mobility disruptions but comparably mild food system disruptions, were expected to result in a 14·3 % rise in the prevalence of moderate or severe wasting among children under the age of five across 118 low- and middle-income countries^(1)^. After COVID-19 was declared a pandemic in March 2020^(3)^, many countries went into partial or full lockdown, including Malaysia, Indonesia, Thailand and Vietnam. During lockdown, governmental support and food assistance programmes were either continued or purposely made available to families in all countries, especially to monetary-poor households. Despite this support, the pandemic still had a negative impact on income stability and perceived stress levels in parents and guardians which might have compromised their ability to take care of their children’s lifestyle, including diet and physical activity^(4)^. At the same time, the outbreak of the pandemic presented an unique opportunity to assess the impact of COVID-19 on parents/guardians and their children, that were already recruited for participation in the South-East Asian Nutrition Surveys II (SEANUTS II) main study.

SEANUTS II is the successor to SEANUTS I, a nationally representative multi-country survey that was conducted in Malaysia, Indonesia, Thailand and Vietnam between 2010 and 2011, which assessed the nutritional status and lifestyle factors of more than 16 500 children aged 0·5–12·9 years old^(5)^. In SEANUTS II, the nutritional status and lifestyle factors of 13 933 children, aged 0·5–12·9 years, have been assessed for the same four countries as SEANUTS I. SEANUTS II was conducted between May 2019 and April 2021. The purpose of SEANUTS II was to continue the monitoring of the nutritional status and lifestyle behaviours of young children in Southeast Asia. After the outbreak of the COVID-19 pandemic, a questionnaire was specifically developed to assess the impact of the COVID-19 pandemic on work status, household expenditures as well as children’s dietary intake and lifestyle behaviours in the SEANUTS II study cohort. This deemed relevant as it had been reported that lifestyle changes in school-aged children, such as increased virtual education and demise of social interactions, can impact nutrition, education and mental health, especially in monetary-poor households, eventually leading to less well-being and suboptimal development^(6,7)^. The aim of this paper is to describe the economic, lifestyle and nutritional impact of the COVID-19 pandemic on parents, guardians and children in Malaysia, Indonesia, Thailand and Vietnam.

## Methods

### Study design

**Fig. 1:**
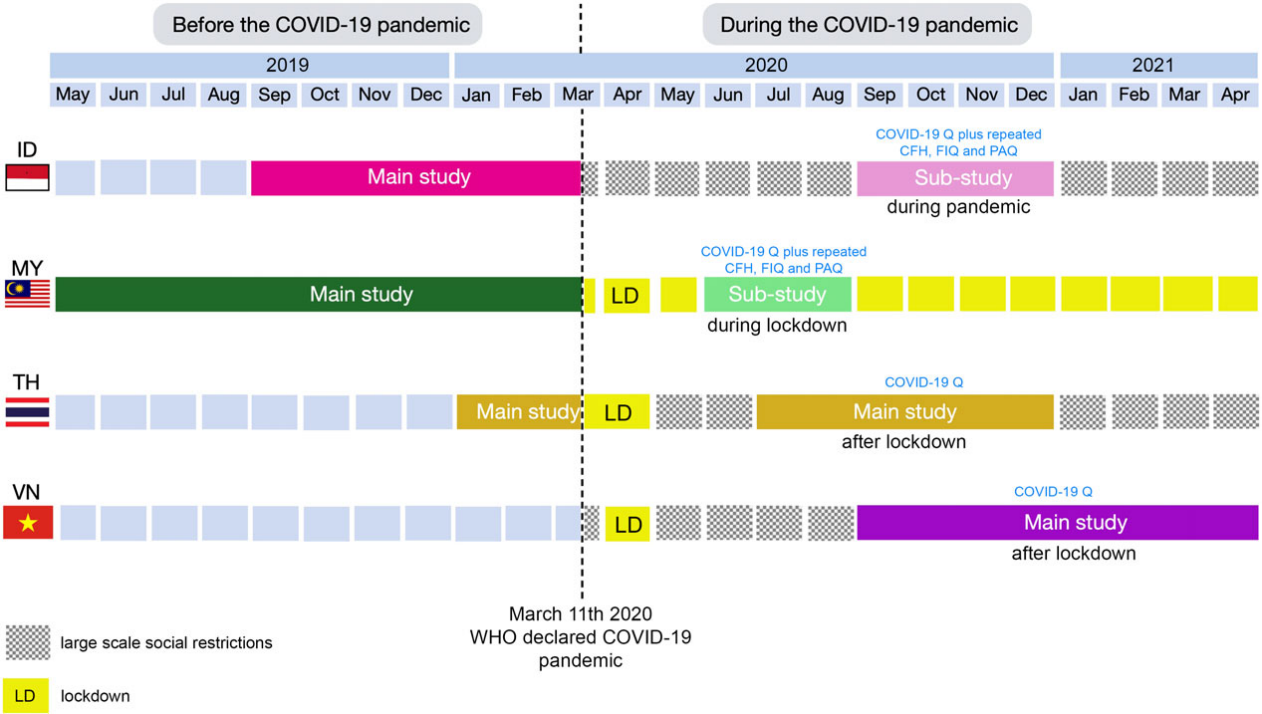
Data collection. For Indonesia and Malaysia, data collection for the SEANUTS II main study was completed before the pandemic and can therefore be regarded as a baseline for the COVID-19 questionnaire, which was administered during the pandemic, and constitutes a genuine sub-study. In Indonesia, the COVID-19 questionnaire was administered as well as repeated CFH, FIQ and PAQ (for specific age groups). In Malaysia, the COVID-19 questionnaire was administered as well as repeated CFH, FIQ and PAQ (for school-aged children). For Thailand, the COVID-19 questionnaire and PAQ (for specific age groups) were administered along with SEANUTS II main study data collection. For Vietnam, the COVID-19 questionnaire, FIQ and PAQ (for specific age groups) were administered along with SEANUTS II main study data collection. For Thailand and Vietnam, the CHF questionnaire was part of the main study but was not repeated. ID: Indonesia, MY: Malaysia, TH: Thailand and VN: Vietnam.

SEANUTS II is a cross-sectional study conducted in four countries: Malaysia, Indonesia, Thailand and Vietnam in both urban and rural areas. Apparently healthy children had to be within the age of 0·5–12 years and citizen of the studied country. Exclusion criteria were physical disability and genetic, cardiovascular or respiratory illness that limited physical activity. In total, the study recruited 13 933 children and their parents/guardians^(8)^. The COVID-19 analysis of SEANUTS II children can be considered a sub-study of the main SEANUTS II study for Malaysia and Indonesia, while it can be considered part of the main SEANUT II study, as it was conducted along with the main study, in Thailand and Vietnam (Fig. 1). Malaysia and Indonesia implemented the COVID-19 questionnaire, after main study data collection was terminated due to the start of the pandemic; in a subgroup of children (∼24 % and ∼43 % of recruited participants in Malaysia and Indonesia, respectively), Thailand implemented the questionnaire in ∼86 % of the children while all children in Vietnam completed it. For data collection, various survey methods were used. Malaysia used online surveys via SurveyMonkey, Indonesia conducted telephone interviews, while Thailand and Vietnam used face-to-face interviews. Data collection in Malaysia took place when schools were not yet open because of lockdown restrictions. In the other countries, children were already going back to school^(8)^.

### SEANUTS II COVID-19 study population

Healthy children and their parents/guardians were included from urban and rural regions in Malaysia, Indonesia, Thailand and Vietnam for the main SEANUTS II study. Children were between 0·5 and 12·9 years except for Vietnamese children who were between 0·5–11·9 years old because primary schools end in Vietnam one year earlier than in the other countries. As for Malaysia and Indonesia, a sub-sample of the already recruited children for the main study were requested to participate in the COVID-19 sub-study, they were therefore a few months older at the time of COVID-19 questionnaire administration.

Children from Malaysia and Indonesia, without information on food intake (Malaysia), changes in intake (Indonesia) or food insecurity during COVID-19 lockdown, were excluded from the analyses. In total, 477 children were excluded, leaving a grand total of 9203 children to be included in the COVID-19 analysis.

### SEANUTS II main study data

Collection of SEANUTS II main study data has been described in detail elsewhere^(8)^. In short, the following measurements are of relevance to this manuscript: (a) socioeconomic and general health status (Socio-Economic Status Questionnaire (SES)), (b) dietary intake and food habits (Child Food Habit questionnaire (CFH))^(4,9)^, covering meal patterns and main food groups, (c) the Food Insecurity Questionnaire (FIQ)^(10,11)^ assessing four levels of food insecurity with increasing severity – food secure, household food insecure, individual or adult food insecure and child hunger. Thailand did not implement the FIQ and (d) physical activity. Physical activity was assessed by a Physical Activity Questionnaire (PAQ)^(12,13)^. Sample size was calculated based on nutritional issues which were of public health relevance per country. Each country used a multi-stage clustered sampling approach based on national population census data. Both urban and rural areas were included in the random selection of enumeration areas^(8)^.

### COVID-19 questionnaire

To understand the impact of the coronavirus pandemic on the economic situation of households, lifestyle and food habits of SEANUTS II children, a COVID-19 questionnaire was specifically developed and implemented in all countries. Malaysia was the first to develop the questionnaire, mainly repeating FIQ^(10,11)^, CFH^(4,9)^ and PAQ^(12,13)^ from the main study as well as adding additional questions specific to the COVID-19 pandemic. Thailand and Indonesia further developed the COVID-19 questionnaire while Vietnam used the same questionnaire as Thailand. The COVID-19 questionnaire consisted of questions addressing (a) the parents’ and/or main guardian’s work status, (b) monthly household income, (c) household food expenditure patterns, (d) children’s dietary intake patterns, such as changes in type of food, portion size and snacks taken, (e) receipt of special governmental or other support during the pandemic and (f) children’s physical activity behaviours and screen time. All questions were self-reported by the parents/guardians. As Malaysia and Indonesia repeated a selection of questions from the main study CFH^(4,9)^, FIQ^(10,11)^ and PAQ^(12,13)^ questionnaires, they could calculate actual changes based on the first and second reporting.

### Study population for analyses

Malaysia was the first to implement the questionnaire in the period of June to August 2020, after SEANUTS II main study data collection was prematurely terminated due to outbreak of the pandemic, to a subgroup of children (*n* 703) from the main SEANUTS II study population (Fig. 1). This corresponds to 24 % of all recruited Malaysian SEANUTS II children. The questionnaire asked for self-reported changes ‘during lockdown’. Besides questions related to COVID-19, Malaysia also repeated a selection of questions from the main study questionnaires CFH^(4,9)^ (*n* 703), FIQ^(10,11)^ (*n* 694) children and PAQ^(12,13)^ (*n* 483), yielding information about possible changes compared to the situation before outbreak of the pandemic. It should be noted that for Malaysia, in contrast to the other countries, no questions about self-reported changes in children’s food intake patterns were included in the COVID-19 questionnaire. Questions on monthly household income were only repeated for Malaysia as household income was one of the questions of the SES questionnaire of the main study for Malaysia.

Thailand was the second country to administer the COVID-19 questionnaire from July to December 2020, alongside execution of the main study (Fig. 1). The questionnaire was administered to 86 % of all Thai participants, that is *n* 3001 participants. As the main study had started before the onset of the pandemic, subjects of which data were collected during this period were not selected for the COVID-19 analysis. Questions were directed at self-reported differences between the periods ‘before COVID-19’ *v*. ‘during COVID-19 lockdown (26 March 2020 until 30 April 2020)’ *v*. ‘after lockdown (but still during the pandemic)’. Thailand also included an additional question about specific school milk programmes^(14)^. A selection of PAQ^(12,13)^ questions was included in the COVID-19 questionnaire.

Indonesia and Vietnam were the last to implement the questionnaire. Indonesia administered the COVID-19 questionnaire to a subgroup (43 %, *n* 1498) of SEANUTS II children in the period from September to December 2020 (Fig. 1). The questionnaire asked for self-reported changes ‘before start COVID-19 pandemic’ *v*. ‘during COVID-19 pandemic’. In addition to this questionnaire, Indonesia also repeated a selection of questions from the main study questionnaires CFH^(4,9)^, FIQ^(10,11)^ and PAQ^(12,13)^, as Malaysia did, allowing for direct comparison with the situation before the outbreak of the pandemic. It should be noted that the repeated CFH questions were only administered to a subset of children (*n* 954) as some children had not filled out the CFH questionnaire in the main study as they were <2 years old at that time or because they could not be reached for the telephone interview.

In contrast to the other three countries, no clear COVID-19 lockdowns were implemented by the Government of Indonesia but instead periods of large-scale social restrictions were implemented^(15)^.

In Vietnam, the COVID-19 questionnaire was administered from September 2020 until April 2021 to all Vietnamese participants (*n* 4001) (Fig. 1). As was the case for Thailand, COVID-19 data collection was running alongside the execution of the main study. Self-reported changes for the following comparisons were made: ‘before start COVID-19 pandemic (before 1 April 2020)’, ‘during COVID-19 lockdown (during 01 April 2020 to 22 April 2020)’ and ‘after lockdown (but still during pandemic)’. A selection of questions from the FIQ^(10,11)^ and PAQ^(12,13)^ questionnaires was included in the COVID-19 questionnaire. Vietnam also included an additional question about availability of school milk.

### Statistical methods

All statistical analyses were performed on unweighted data. A binomial test was used to assess if the percentage of self-reported increase was different from the percentage of self-reported decrease. A McNemar test was used to test if the self-reported changes during lockdown differed from the self-reported changes after lockdown. For the repeated measurements, a Wilcoxon signed ranked test was used to assess if the change before and during lockdown/pandemic was significant. Generalised estimating equations were used to examine if change was different between rural and urban areas. Statistical analyses were performed using IBM SPSS Statistics version 23·0 for Windows (IBM Corp.). Throughout the study, a *P*-value <0·05 indicates statistical significance.

## Results

### Baseline data

Subject characteristics of those participating in the COVID-19 study can be found in Table 1. The proportion of children from rural and urban areas in Indonesia was very similar (*n* 754 and *n* 744, respectively), whereas for Thailand and Vietnam most children came from rural regions (*n* 2063 and *n* 2787, respectively). In contrast, in Malaysia, most study participants came from urban areas (*n* 512 *v*. *n* 191 rural). The ratio of ‘male-female’ was very similar across countries as well as across urban and rural areas. Of the total study population, 1498 children came from Indonesia (754 (50·3 %) females and 744 (49·7 %) males), 703 from Malaysia (361 (51·4 %) females and 342 (48·6 %) males), 3001 from Thailand (1497 (49·9 %) females and 1504 (50·1 %) males), and 4001 from Vietnam (1981 (49·5 %) females and 2020 (50·5 %) males). Stunting was most prevalent in Indonesia (27·9 % rural and 18·1 % urban), as well as the percentage of young children (<5 years old) with underweight (20·2 % rural and 18·5 % urban). The percentage of overweight and obese children was highest in urban Vietnam (15·4 % and 14·3 %, respectively) and rural Malaysia (13·1 % and 11·5 %, respectively).

**Table 1:** Baseline data of COVID-19 study participants

	Indonesia				Malaysia				Thailand				Vietnam			
	Before pandemic				Before pandemic				After lockdown				After lockdown			
	Rural (*n* 754)		Urban (*n* 744)		Rural (*n* 191)		Urban (*n* 512)		Rural (*n* 2063)		Urban (*n* 938)		Rural (*n* 2787)		Urban (*n* 1214)	
Data collection period	Mean	sd	Mean	sd	Mean	sd	Mean	sd	Mean	sd	Mean	sd	Mean	sd	Mean	sd
Age (years)	4·2	3·3	4·8	3·6	7·8	2·9	7·1	2·8	5·4	3·6	5·3	3·5	5·5	3·4	5·5	3·3
	*n*	%	*n*	%	*n*	%	*n*	%	*n*	%	*n*	%	*n*	%	*n*	%
Male (%)	387	51·3	357	48·0	89	46·6	253	49·4	1044	50·5	460	49·0	1404	50·4	616	50·7
Stunted (%)	210	27·9	135	18·1	20	10·5	36	7·0	118	5·7	42	4·5	337	12·1	74	6·1
Underweight <5 years (%)	101	20·2	84	18·5	3	1·6	9	1·8	45	4·1	21	4·1	115	9·0	25	4·5
Overweight (%)	25	3·3	36	4·8	25	13·1	42	8·2	169	8·3	85	9·1	255	9·5	184	15·4
Obesity (%)	19	2·5	21	2·8	22	11·5	41	8·0	171	8·4	80	8·6	201	7·5	171	14·3
Father’s education																
Non-schooling/Primary school (%)	224	29·7	118	16·8	10	5·6	13	2·6	432	24·3	172	20·9	337	12·5	108	9·1
Secondary school (%)	460	61·0	504	71·7	98	54·4	234	47·4	1029	57·8	470	57·1	1731	64·1	562	47·2
Tertiary school (%)	49	6·5	81	11·5	72	40·0	247	50·0	318	17·9	181	22·0	632	23·4	521	43·7
Mother’s education																
Non-schooling/Primary School (%)	204	27·4	128	17·3	1	0·5	11	2·2	381	19·6	121	13·4	383	13·9	109	9·0
Secondary school (%)	477	64·0	522	70·6	98	52·1	202	39·8	1130	58·2	534	59·2	1641	59·7	537	44·5
Tertiary School (%)	64	8·6	89	12·0	89	47·3	295	58·1	432	22·2	247	27·4	724	26·3	562	46·5
Physical activity (>7 years; days per week)																
Days per week in which the child is occasionally or frequently active	0·5	0·5	0·5	0·5	2·3	2·5	2·0	2·2	1·7	2·3	1·6	2·3	2·3	2·8	2·3	2·9
	Median	IQR	Median	IQR	Median	IQR	Median	IQR	Median	IQR	Median	IQR	Median	IQR	Median	IQR
Food groups																
Basic foods																
Vegetables (times per day)	0·4	0·3–0·9	0·6	0·3–1·0	0·4	0·1–0·7	0·6	0·3–0·9	0·6	0·3–1·0	0·6	0·3–1·0	0·9	0·4–1·0	1·0	0·4–1·0
Fruits (times per day)	0·4	0·3–0·9	0·4	0·1–0·6	0·4	0·3–0·6	0·4	0·3–0·6	0·6	0·3–0·9	0·6	0·4–0·9	0·6	0·4–1·0	0·9	0·4–1·0
Fish (times per day)	0·1	0·0–0·3	0·4	0·1–0·9	0·6	0·3–0·9	0·4	0·1–0·7	0·6	0·3–1·0	0·6	0·3–0·9	0·0	0·0–0·1	0·0	0·0–0·1
Eggs (times per day)	0·7	0·4–1	0·7	0·4–1·0	0·4	0·1–0·7	0·4	0·4–0·7	0·7	0·4–1·0	0·7	0·4–1·0	0·7	0·4–1·0	0·4	0·4–0·4
Milk (250 ml serves/d)	0·3	0·0–0·4	0·3	0·0–0·6	0·3	0·0–0·7	0·3	0·0–0·7	1·1	0·9–2·0	1·1	0·9–2·0	0·6	0·0–1·3	0·7	0·0–1·6
YCF (250 ml serves/d; <4 years)	0·0	0·0–0·0	0·06	0–1·2	0·0	0·0–1	0·8	0·03–1·2	0·0	0·0–0·0	0·0	0·0–0·0	0·0	0·0–0·0	0·0	0·0–0·4
Non-basic & convenience food (times per day)																
Deep fried food	1·0	0·4–1·0	1	0·6–1·0	0·6	0·4–0·9	0·4	0·3–0·7	0·6	0·4–0·9	0·6	0·4–0·9	0·3	0·1–0·4	0·3	0·1–0·4
Local cakes (*kuih*)	0·3	0·1–0·4	0·3	0·1–0·6	0·4	0·3–0·6	0·3	0·1–0·6	0·0	0·0–0·3	0·1	0·0–0·3	0·1	0·0–0·3	0·1	0·0–0·3
Confectionery	0·6	0·3–1·0	0·6	0·3–1·0	0·4	0·3–0·6	0·3	0·1–0·4	0·4	0·3–0·9	0·6	0·3–0·9	0·3	0·1–0·6	0·3	0·1–0·4
Sugar-sweetened beverages	0·4	0·0–1·0	0·4	0·1–1·0	0·4	0·3–1·0	0·3	0·1–0·6	0·4	0·3–0·9	0·4	0·3–0·7	0·1	0·0–0·3	0·0	0·0–0·1

HAZ: Height-for-Age Z-score; WAZ: weight-for-Age Z-score; BAZ: BMI-for-Age Z-score

Data are reported as mean (s d), median (Q1–Q3) or *n* (%). Milk includes fresh milk and milk powder, flavoured milk and evaporated milk. Overweight was defined as BAZ > 2 sd to ≤ 3 sd for children younger than 5 years; BAZ > 1 sd to ≤ 2 sd for children older than 5 years. Obesity was defined as BAZ > 3 sd for children younger than 5 years; BAZ > 2 sd for children older than 5 years. Stunted was defined as HAZ < –2 sd. Underweight was defined as WAZ < –2 sd. YCF: young child formula. Except for anthropometry, all data are based on questionnaires^(8)^.

In all countries, secondary schooling was the most common education status of parents/guardians. The proportion of tertiary schooling was highest in urban Malaysian fathers (50·0 %) and urban Malaysian mothers (58·1 %) followed by urban Vietnamese (46·5 %) mothers.

At baseline, older children (>7 years) were physically most active in Malaysia and Vietnam.

Based on the main study’s first CFH questionnaire, baseline intake of vegetables and fruits by SEANUTS II children was highest in Vietnam and especially in urban Vietnam. Baseline fish intake was lowest in Vietnam, and baseline milk intake was highest in Thailand. The baseline intake of non-basic and convenience foods was largely comparable across countries with the highest intake of deep-fried foods in Indonesia and the lowest intake of sugar-sweetened beverages in Vietnam.

### Socioeconomic impact of the pandemic

The COVID-19 pandemic significantly affected the socioeconomic situation of families in the various countries (Table 2). Monthly household income decreased significantly in all countries for most families. Proportions of decrease ranged from 39·2 % (urban Vietnam) to 78·7 % (urban Thailand). Especially for Thailand, the decrease in income was highly significant.

**Table 2:** Change in socioeconomic parameters compared to before the pandemic

	Indonesia (ID)				Malaysia (MY)				Thailand (TH)				Vietnam (VN)			
	Rural (*n* 754)		Urban (*n* 744)		Rural (*n* 191)		Urban (*n* 512)		Rural (*n* 2063)		Urban (*n* 938)		Rural (*n* 2787)		Urban (*n* 1214)	
	*n*	%	*n*	%	*n*	%	*n*	%	*n*	%	*n*	%	*n*	%	*n*	%
Monthly household income during lockdown^*^ (MY^†^, TH and VN) or during pandemic (ID)^*^																
Decrease (%)	558	74·0	512	68·8	109	61·2	307	64·4	1612	78·1	738	78·7	1263	45·3	476	39·2
Increase (%)	16	2·1	13	1·7	33	18·5	88	18·4	11	0·5	5	0·5	14	0·5	6	0·5
Same (%)	180	23·9	219	29·4	36	20·2	82	17·2	440	21·3	195	20·8	1510	54·2	732	60·3
*P*-value	<0·001		<0·001		<0·001		<0·001		<0·001		<0·001		<0·001		<0·001	
Monthly household income after lockdown^*^																
Decrease (%)	NA		NA		NA		NA		926	44·9	403	43·0	241	8·6	46	3·8
Increase (%)	NA		NA		NA		NA		34	1·6	10	1·1	236	8·5	113	9·3
Same (%)	NA		NA		NA		NA		1103	53·5	525	56·0	2310	82·9	1055	86·9
*P*-value									<0·001		<0·001		0·855		<0·001	
Monthly household income spent on food during lockdown^*^ (MY^†^, TH, VN) or during pandemic (ID)^*^																
Decrease (%)	319	42·3	250	33·6	62	34·1	148	31·2	596	28·9	294	31·3	520	18·7	151	12·4
Increase (%)	145	19·2	169	22·7	98	53·8	254	53·6	313	15·2	124	13·2	55	2·0	27	2·2
Same (%)	290	38·5	325	43·7	22	12·1	72	15·2	1154	55·9	520	55·4	2212	79·4	1036	85·3
*P*-value	<0·001		<0·001		0·005		<0·001		<0·001		<0·001		<0·001		<0·001	
Monthly household income spent on food after lockdown^*^																
Decrease (%)	NA		NA		NA		NA		347	16·8	125	13·3	130	4·7	22	1·8
Increase (%)	NA		NA		NA		NA		131	6·3	50	5·3	68	2·4	32	2·6
Same (%)	NA		NA		NA		NA		1585	76·8	763	81·3	2589	92·9	1160	95·6
*P*-value									<0·001		<0·001		<0·001		0·220	
Loss of job father or male guardian^‡^																
Before pandemic/lockdow*n* (%)	0	0·0	4	0·5	NA		NA		4	0·2	2	0·2	10	0·4	5	0·4
During pandemic/lockdow*n* (%)	31	4·1	36	4·8	6	3·2	20	3·9	141	6·8	55	5·9	104	3·7	64	5·3
After lockdown (%)	NA		NA		NA		NA		29	1·4	6	0·6	16	0·6	7	0·6
*P*-value (before-during)	<0·001		<0·001						<0·001		<0·001		<0·001		<0·001	
* P*-value (before-after)									<0·001		0·219		0·238		0·754	
Loss of job mother or female guardian^‡^																
Before pandemic/lockdow*n* (%)	0	0·0	0	0·0	NA		NA		3	0·1	1	0·1	9	0·3	3	0·2
During pandemic/lockdow*n* (%)	17·0	2·3	22·0	3·0	2·0	1·0	10·0	2·0	134	6·5	61	6·5	70	2·5	48	4·0
After lockdown (%)	NA		NA		NA		NA		36	1·7	12	1·3	12	0·4	1	0·1
* P*-value (before-during)	<0·001		<0·001						<0·001		<0·001		<0·001		<0·001	
*P*-value (before-after)									<0·001		0·001		0·607		0·625	
Work from home father or male guardian^‡^																
Before pandemic/lockdow*n* (%)	62	8·2	40	5·4	NA		NA		92	4·5	39	4·2	445	16·0	194	16·0
During pandemic/lockdow*n* (%)	76	10·1	64	8·6	60	31·4	193	37·7	99	4·8	49	5·2	751	26·9	329	27·1
After lockdown (%)	NA		NA		NA		NA		97	4·7	39	4·2	454	16·3	199	16·4
*P*-value (before-during)	0·003		<0·001						0·349		0·031		<0·001		<0·001	
*P*-value (before-after)									0·302		1·000		0·176		0·180	
Work from home mother or female guardian^‡^																
Before pandemic/lockdow*n* (%)	82	10·9	113	15·2	NA		NA		131	6·3	53	5·7	728	26·1	314	25·9
During pandemic/lockdow*n* (%)	98	13·0	124	16·7	68	35·6	204	39·8	133	6·4	61	6·5	1048	37·6	448	36·9
After lockdown (%)	NA		NA		NA		NA		130	6·3	53	5·7	742	26·6	317	26·1
*P*-value (before-during)	0·006		0·063						0·907		0·280		<0·001		<0·001	
*P*-value (before-after)									1·000		1·000		0·045		0·581	

NA: not available.

Data are reported as *n* (%).

* Statistical tests: %increase = %decrease: binominal test (Ho is no overall change. No overall change is defined as participants having answered ‘the same’ or the number of participants answering ‘increase’ equalled the number of participants answering ‘decrease’).

† Statistical tests: For Malaysia, impact of COVID-19 on household income and household income spent on food is based on parents self-reporting twice, that is before the pandemic and during lockdown (part of SES questionnaire). The change was calculated based on the first and second reporting.

‡ Statistical tests: ‘during pandemic’ *v.* ‘before pandemic’ (Indonesia) and ‘during COVID-19 lockdown’ *v*. ‘before pandemic’ (Malaysia) or ‘before pandemic’ *v*. ‘during COVID-19 lockdown’ or ‘before pandemic’ *v*. ‘after COVID-19 lockdown’ (both for Vietnam and Thailand): McNemar test.

In all countries, except Malaysia, food expenditure decreased or remained stable during lockdown. In Malaysia, food expenditure actually increased in both rural (53·8 %) and urban (53·6 %) regions. For Thailand and Vietnam, the percentage of households reporting decreased food expenditure was smaller after lockdown than during lockdown. For Indonesia and Malaysia, no ‘after lockdown’ data were collected.

During the pandemic and lockdowns, the number of parents/guardians losing their jobs increased significantly compared to the situation pre-pandemic in all countries. This was seen for both fathers/ male guardians and mothers/female guardians.

The situation of parents/guardians that were working from home changed less consistently during the pandemic/lockdowns across the countries. The number of mothers/female guardians working from home increased significantly due to the pandemic in rural Indonesia. This was also seen in rural and urban Malaysia and Vietnam during lockdown but not in Thailand. Interestingly, this significant increase persisted after lockdown in rural Vietnam. The number of fathers and male guardians working from home increased significantly during the pandemic in rural and urban areas of Indonesia. Also, in rural and urban areas of Malaysia and Vietnam, as well as in urban Thailand, this increase was seen when comparing the period during lockdown with the time before the pandemic (Table 2). The largest quantitative changes in work status were observed in Malaysia. Overall, during lockdown in Malaysia, the percentage of fathers/male guardians not working increased from 4 % to 27 %, those working at the office decreased from 96 % to 29 % while 36 % of them were working from home. For mothers/female guardians, those not working increased from 33 % to 40 %, those working at the office decreased from 65 % to 14 % while 39 % of them were working from home.

### Self-reported changes in food intake patterns

Data on self-reported changes in food intake (based on questions in the COVID-19 questionnaire) during pandemic/lockdown were available for SEANUTS II children from Indonesia (during pandemic *v*. before pandemic), Thailand and Vietnam (during lockdown *v*. before pandemic) (Table 3a). Intake of food groups was not different before the pandemic when compared to during pandemic/lockdown as reported by ∼60·0 to ∼95·0 % of all households. Most households did not dramatically change their food habits during the pandemic. This does not imply that there were no changes at all. When comparing the % increase to the % decrease of the various food groups in the countries, several significant changes were identified. In rural Indonesia, most of the children that changed their food intake during the pandemic decreased their consumption of vegetables (*P*-value < 0·001), fruits (*P*-value < 0·001), meat/poultry/seafood (*P*-value < 0·001), eggs (*P*-value 0·002), milk (*P*-value < 0·001), other dairy products (*P*-value < 0·001), canned foods (*P*-value < 0·001), convenience food (*P*-value < 0·001), processed foods (*P*-value < 0·001), sweetened beverages (*P*-value < 0·001) and snacks (*P*-value 0·007) while they increased their consumption of rice/cereals (*P*-value < 0·001). Most of the children also increased the portion size of their main meals (*P*-value < 0·001). In urban regions of Indonesia, most of the children that changed their food intake decreased their intake of fruits (*P*-value < 0·001), meat/poultry/seafood (*P*-value < 0·001), other dairy products (*P*-value < 0·001), canned foods (*P*-value 0·012), convenience food (*P*-value < 0·001), processed foods (*P*-value < 0·001), sweetened beverages (*P*-value < 0·001) and snacks (*P*-value < 0·001). Interestingly, portion size of main meals (*P*-value < 0·001) increased. For rural Thailand, the consumption of vegetables (*P*-value 0·022), other dairy products (*P*-value < 0·001), canned foods (*P*-value 0·002), processed foods (p-value 0·005), sweetened beverages (<0·001) all decreased while the consumption of eggs (*P*-value < 0·001), milk (*P*-value 0·013), rice/cereals (*P*-value 0·001) and portion size of main meals (*P*-value < 0·001) increased. In urban regions of Thailand, only the intake of sweetened beverages (0·003) decreased while the intake of eggs (*P*-value 0·001), milk (*P*-value 0·020) and portion size of main meals (*P*-value 0·010) all increased. Finally, in rural areas of Vietnam, the majority of children that changed their food intake during the pandemic decreased their intake of vegetables, fruits, meat/poultry/seafood, eggs, milk, young child formula, other dairy products, rice/cereals, canned foods, convenience food, processed foods, sweetened beverages and snacks (*P*-value < 0·001 for all). Also portion size of main meals (*P*-value < 0·001) decreased. In urban Vietnam, the majority of children that changed their food intake decreased consumption of meat/poultry/seafood (*P*-value 0·021), milk (*P*-value 0·017), young child formula (*P*-value 0·004), other dairy products (*P*-value 0·001), canned foods (*P*-value < 0·001), convenience food (*P*-value 0·001), processed foods (*P*-value < 0·001), sweetened beverages (*P*-value < 0·001) and snacks (*P*-value < 0·001). These children also decreased their portion size of main meals (*P*-value 0·029).

**Table 3a:** Self-reported changes in foods consumed by SEANUTS II children ‘during pandemic’ (Indonesia) and ‘during COVID-19 lockdown’ (Thailand and Vietnam) *v*. ‘before pandemic’

	Indonesia				Thailand				Vietnam			
	Rural (*n* 754)		Urban (*n* 745)		Rural (*n* 2063)		Urban (*n* 938)		Rural (*n* 2787)		Urban (*n* 1214)	
	*n*	%	*n*	%	*n*	%	*n*	%	*n*	%	*n*	%
Basic foods												
Vegetables and fruits												
Vegetables												
Decrease (%)	171	22·7	125	16·8	68	3·3	23	2·5	146	5·2	28	2·3
Increase (%)	108	14·3	103	13·8	43	2·1	13	1·4	21	0·8	15	1·2
Same (%)	475	63·0	517	69·4	1831	88·8	851	90·7	2620	94·0	1171	96·5
Do not eat (%)					121	5·9	51	5·4	0	0·0	0	0·0
*P*-value	<0·001		0·164		0·022		0·132		<0·001		0·066	
Fruits												
Decrease (%)	182	24·1	165	22·2	72	3·5	27	2·9	166	6·0	30	2·5
Increase (%)	102	13·5	94	12·6	57	2·8	18	1·9	20	0·7	17	1·4
Same (%)	470	62·3	486	65·2	1847	89·5	859	91·6	2601	93·3	1167	96·1
Do not eat (%)					87	4·2	34	3·6	0·0	0·0	0	0·0
*P*-value	<0·001		<0·001		0·218		0·233		<0·001		0·079	
Protein-rich foods												
Meat/poultry/seafood												
Decrease (%)	230	30·5	176	23·7	69	3·3	21	2·2	158	5·7	33	2·7
Increase (%)	78	10·3	77	10·3	62	3·0	19	2·0	34	1·2	16	1·3
Same (%)	446	59·2	491	66·0	1850	89·7	868	92·5	2595	93·1	1165	96·0
Do not eat (%)					82	4·0	30	3·2	0	0·0	0	0·0
*P*-value	<0·001		<0·001		0·600		0·875		<0·001		0·021	
Eggs												
Decrease (%)	169	22·4	118	15·9	26	1·3	10	1·1	126	4·5	26	2·1
Increase (%)	116	15·4	89	12·0	81	3·9	31	3·3	32	1·1	16	1·3
Same (%)	469	62·2	538	72·2	1885	91·4	871	92·9	2629	94·3	1172	96·5
Do not eat (%)					71	3·4	26	2·8	0	0·0	0	0·0
*P*-value	0·002		0·051		<0·001		0·001		<0·001		0·164	
Milk												
Decrease (%)	109	14·5	75	10·1	50	2·4	13	1·4	125	4·5	28	2·3
Increase (%)	41	5·4	65	8·7	79	3·8	29	3·1	28	1·0	12	1·0
Same (%)	604	80·1	604	81·2	1807	87·6	829	88·4	2634	94·5	1174	96·7
Do not eat (%)					127	6·2	67	7·1	0	0·0	0	0·0
*P*-value	<0·001		0·447		0·013		0·020		<0·001		0·017	
Young child formula												
Decrease (%)	90	11·9	71	9·5	8	0·4	6	0·6	127	4·6	30	2·5
Increase (%)	43	5·7	66	8·9	10	0·5	6	0·6	19	0·7	11	0·9
Same (%)	621	82·4	607	81·6	283	13·7	110	11·7	2641	94·8	1173	96·6
Do not eat (%)					1762	85·4	816	87·0	0	0·0	0	0·0
*P*-value	0·512		0·733		0·815		1·000		<0·001		0·004	
Other dairy products												
Decrease (%)	61	8·1	53	7·1	51	2·5	12	1·3	136	4·9	35	2·9
Increase (%)	17	2·3	18	2·4	23	1·1	5	0·5	17	0·6	11	0·9
Same (%)	676	89·7	673	90·5	1480	71·7	695	74·1	2634	94·5	1168	96·2
Do not eat (%)					509	24·7	226	24·1	0	0·0	0	0·0
*P*-value	<0·001		<0·001		0·002		0·143		<0·001		0·001	
Carbohydrate-rich food												
Rice/cereals												
Decrease (%)	111	14·7	76	10·2	34	1·6	15	1·6	110	3·9	23	1·9
Increase (%)	122	16·2	91	12·2	70	3·4	27	2·9	33	1·2	12	1·0
Same (%)	521	69·1	577	77·6	1895	91·9	872	93·0	2644	94·9	1179	97·1
Do not eat (%)					64	3·1	24	2·6	0	0·0	0	0·0
*P*-value	<0·001		0·279		0·001		0·088		<0·001		0·090	
Non-basic/convenience foods												
Canned foods												
Decrease (%)	63	8·4	50	6·7	54	2·6	18	1·9	129	4·6	38	3·1
Increase (%)	8	1·1	27	3·6	26	1·3	10	1·1	18	0·6	11	0·9
Same (%)	683	90·6	667	89·7	1436	69·6	635	67·7	2640	94·7	1165	96·0
Do not eat (%)					547	26·5	275	29·3	0	0·0	0	0·0
*P*-value	**<**0·001		0·012		0·002		0·185		<0·001		<0·001	
Convenience food												
Decrease (%)	155	20·6	136	18·3	51	2·5	18	1·9	126	4·5	36	3·0
Increase (%)	28	3·7	43	5·8	53	2·6	25	2·7	19	0·7	12	1·0
Same (%)	571	75·7	565	75·9	1575	76·3	710	75·7	2642	94·8	1166	96·0
Do not eat (%)					384	18·6	185	19·7	0	0·0	0	0·0
*P*-value	<0·001		<0·001		0·922		0·360		<0·001		0·001	
Processed foods												
Decrease (%)	125	16·6	130	17·5	65	3·2	27	2·9	136	4·9	39	3·2
Increase (%)	38	5·0	26	3·5	36	1·7	14	1·5	17	0·6	11	0·9
Same (%)	591	78·4	588	79·0	1730	83·9	802	85·5	2634	94·5	1164	95·9
Do not eat (%)					232	11·2	95	10·1	0	0·0	0	0·0
*P*-value	<0·001		<0·001		0·005		0·060		<0·001		<0·001	
Sweetened beverages												
Decrease (%)	156	20·7	127	17·1	94	4·6	37	3·9	143	5·1	43	3·5
Increase (%)	76	10·1	43	5·8	44	2·1	15	1·6	18	0·6	11	0·9
Same (%)	522	69·2	574	77·2	1665	80·7	771	82·2	2626	94·2	1160	95·6
Do not eat (%)					260	12·6	115	12·3	0	0·0	0	0·0
*P*-value	<0·001		<0·001		<0·001		0·003		<0·001		<0·001	
Snacks												
Decrease (%)	170	22·5	165	22·2	111	5·4	44	4·7	147	5·3	49	4·0
Increase (%)	123	16·3	103	13·8	84	4·1	34	3·6	21	0·8	13	1·1
Same (%)	461	61·1	476	64·0	1717	83·2	784	83·6	2619	94·0	1152	94·9
Do not eat (%)					151	7·3	76	8·1	0	0·0	0	0·0
*P*-value	0·007		<0·001		0·062		0·308		<0·001		<0·001	
Others												
Portion size of main meals												
Decrease (%)	100	13·3	77	10·3	60	2·9	28	3·0	92	3·3	25	2·1
Increase (%)	185	24·5	174	23·4	133	6·4	52	5·5	34	1·2	11	0·9
Same (%)	469	62·2	493	66·3	1870	90·6	858	91·5	2661	95·5	1178	97·0
Do not eat (%)												
*P*-value	<0·001		<0·001		<0·001		0·010		<0·001		0·029	

Data are reported as *n* (%). Statistical test: %increase = %decrease with a binominal test.

### Repeated measures analysis of food intake

Both, Indonesia and Malaysia, repeated the CFH questionnaire to assess changes in intake frequencies of foods consumed during pandemic/lockdown as compared to before the pandemic (Table 3b). For Indonesia, significant differences were found for the intake of vegetables, deep-fried foods and sugar-sweetened beverages. The frequency of vegetable intake per week increased in both rural and urban regions (*P*-value < 0·001) while the consumption of deep-fried foods significantly increased in urban areas (*P*-value 0·013) and the consumption of sugar-sweetened beverages significantly decreased in urban areas (*P*-value 0·001) during the pandemic. Significant differences in consumption between Indonesian rural and urban areas were found for fish consumption (*P*-value 0·050), deep-fried foods (*P*-value 0·043) and sugar-sweetened beverages (*P*-value 0·009). In Malaysia, the frequency of vegetables, fruits and eggs consumption significantly increased in both rural (*P*-values 0·007, 0·003, <0·001, respectively) and urban areas (*P*-value < 0·001 and <0·001, <0·001, respectively) during lockdown. The consumption of confectionery by urban children slightly increased (*P*-value 0·009). The consumption of milk decreased in both rural (*P*-value 0·004) and urban (*P*-value 0·001) areas during COVID-19 lockdown. Also, the consumption of sugar-sweetened beverages (rural and urban, *P*-values 0·002 and <0·001) and local cakes (*kuih*) (urban, *P*-value 0·001) significantly decreased.

**Table 3b:** Changes in intake frequencies of foods consumed by SEANUTS II children ‘during pandemic’ *v.* ‘before start pandemic’ in Indonesia and ‘during COVID-19 lockdown’ *v.* ‘before pandemic’ in Malaysia (based on repeated CFH questionnaire)

	Indonesia											Malaysia										
	Rural (*n* 465)					Urban (*n* 489)						Rural (*n* 191)					Urban (*n* 512)					
	Before pandemic		During pandemic			Before pandemic		During pandemic				Before pandemic		During LD			Before pandemic		During LD			
	*n*	%	*n*	%	*P* ^*^	*n*	%	*n*	%	*P* ^*^	*P* ^†^	*n*	%	*n*	%	*P* ^*^	*n*	%	*n*	%	*P* ^*^	*P* ^†^
Basic foods																						
Vegetables																						
Less than once a week/never	0	0·0	0	0·0	<0·001	1	0·3	0	0·0	<0·001	0·971	33	17·5	30	15·9	0·007	63	12·5	58.	11·5	<0·001	0·931
Once a week	60	15·0	27	6·8		58	14·6	31	7·8			21	11·1	13	6·9		48	9·5	34	6·7		
2–3 times/week	307	76·8	320	80·0		300	75·6	314	79·1			59	31·2	55	29·1		127	25·2	114	22·6		
4–6 times/week	33	8·3	53	13·3		38	9·6	52	13·1			44	23·3	35	18·5		143	28·4	94	18·7		
At least once/d	0	0·0	0	0·0		0	0·0	0	0·0			32	16·9	56	29·6		123	24·4	204	40·5		
Fruits																						
Less than once a week/never	20	4·8	25	6·0	0·367	31	7·5	25	6·0	0·209	0·172	9	4·7	20	10·5	0·003	33	6·6	47·0	9·3	<0·001	0·481
Once a week	54	13·0	51	12·3		78	18·8	51	12·3			31	16·3	17	8·9		68	13·5	40	8·0		
2–3 times/week	190	45·7	208	50·1		170	41·0	208	50·1			90	47·4	62	32·6		219	43·5	160	31·8		
4–6 times/week	60	14·4	52	12·5		60	14·5	52	12·5			40	21·1	41	21·6		142	28·2	105	20·9		
At least once/d	92	22·1	79	19·0		76	18·3	79	19·0			20	10·5	50	26·3		41	8·2	151	30·0		
Protein-rich foods																						
Fish																						
Less than once a week/never	222	49·9	230	51·7	0·118	119	28·7	111	26·7	0·226	0·050	NA		NA		NA	NA		NA		NA	NA
Once a week	60	13·5	64	16·2		49	11·8	40	9·6			NA		NA		NA	NA		NA		NA	NA
2–3 times/week	110	24·7	102	22·9		112	27·0	128	30·8			NA		NA		NA	NA		NA		NA	NA
4–6 times/week	42	9·4	36	8·1		97	23·4	77	18·6			NA		NA		NA	NA		NA		NA	NA
At least once/d	11	2·5	5	1·1		38	9·2	59	14·2			NA		NA		NA	NA		NA		NA	NA
Eggs																						
Less than once a week/never	34	7·6	18	4·0	0·079	29	6·3	27·0	5·8	0·920	0·203	10	5·5	14·0	7·7	<0·001	32	6·5	16	3·2	<0·001	0·727
Once a week	49	11·0	38	8·5		23	5·0	24·0	5·2			46	25·1	8	4·4		79	15·9	26	5·2		
2–3 times/week	111	24·9	137	30·8		164	35·5	156·0	33·8			69	37·7	57	31·1		198	39·9	138	27·8		
4–6 times/week	120	27·0	114	25·6		105	22·7	124·0	26·8			44	24·0	58	31·7		135	27·2	164	33·1		
At least once/d	131	29·4	138	31·0		141	30·5	131·0	28·4			14	7·7	46	25·1		52	10·5	152	30·6		
Milk																						
Less than once a week/never	173	37·2	197	42·4	0·159	131	26·8	161	32·9	0·309	0·916	36	19	51	27	0·004	65	12·8	78	15·4	0·001	0·575
Once a week	6	1·3	6	1·3		6	1·2	7	1·4			15	7·9	17	9		18	3·6	36	7·1		
2–3 times/week	51	11·0	35	7·5		83	17·0	45	9·2			36	19	35	18·5		63	12·5	80	15·8		
4–6 times/week	54	11·6	53	11·4		78	16·0	70	14·3			24	12·7	26	13·8		84	16·6	73	14·4		
At least once/d	181	38·9	174	37·4		191	39·1	206	42·1			78	41·3	60	31·7		276	54·5	239	47·2		
Non-basic/ convenience Foods																						
Deep fried foods																						
Less than once a week/never	10	2·3	11	2·6	0·425	2	0·4	1	0·2	0·013	0·043	2	1·1	5	2·6	0·107	12	2·4	23	4·6	0·218	0·393
Once a week	25	5·8	17	4·0		15	3·2	7	1·5			13	6·8	16	8·4		64	12·7	63	12·5		
2–3 times/week	86	20·1	105	24·5		100	21·5	88	18·9			60	31·6	53	27·9		200	39·6	205	40·6		
4–6 times/week	60	14·0	59	13·8		75	16·1	63	13·5			66	34·7	84	44·2		154	30·5	132	26·1		
At least once/d	247	57·7	236	55·1		274	58·8	307	65·9			49	25·8	32	16·8		75	14·9	82	16·2		
Sugar-sweetened beverages																						
Less than once a week/never	100	24·3	102	24·8	0·857	92	22·2	125	30·1	0·001	0·009	22	11·6	42	22·2	0·002	88	17·5	143	28·4	<0·001	0·613
Once a week	49	11·9	29	7·0		46	11·1	40	9·6			21	11·1	22	11·6		79	15·7	73	14·5		
2–3 times/week	110	26·7	134	32·5		100	24·1	102	24·6			57	30·2	49	25·9		174	34·6	155	30·8		
4–6 times/week	52	12·6	48	11·7		44	10·6	55	13·3			38	20·1	43	22·8		83	16·5	78	15·5		
At least once/d	101	24·5	99	24·0		133	32·0	93	22·4			51	27·0	33	17·5		79	15·7	54	10·7		
Local cakes (kuih)																						
Less than once a week/never	96	25·2	101	26·5	0·346	91	23·5	86	22·2	0·193	0·559	13	6·9	16	8·5	0·146	88	17·7	123	24·8	0·001	0·536
Once a week	67	17·6	51	13·4		48	12·4	33	8·5			31	16·4	35	18·5		99	20·0	90	18·1		
2–3 times/week	144	37·8	146	38·3		140	36·1	147	37·9			78	41·3	84	44·4		189	38·1	188	37·9		
4–6 times/week	45	11·8	40	10·5		45	11·6	54	13·9			55	29·1	43	22·8		90	18·1	71	14·3		
At least once/d	29	7·6	43	11·3		64	16·5	68	17·5			12	6·3	11	5·8		30	6·0	24	4·8		
Confectionery																						
Less than once a week/never	33	7·7	33	7·7	0·290	18	4·2	30	6·9	0·604	0·086	10	5·2	11	5·8	0·108	31	6·2	40	8·0	0·009	0·982
Once a week	44	10·2	44	10·2		57	13·2	36	8·3			29	15·2	34	17·8		109	21·8	97	19·4		
2–3 times/week	126	29·2	114	26·5		120	27·8	128	29·6			82	42·9	92	48·2		199	39·7	245	48·9		
4–6 times/week	87	20·2	76	17·6		74	17·1	93	21·5			52	27·2	43	22·5		112	22·4	97	19·4		
At least once/d	141	32·7	164	38·1		163	37·7	145	33·6			18	9·4	11	5·8		50	10·0	22	4·4		

CFH: Child Food Habit. NA: not available. LD: lockdown.

Data are reported as *n* (%).

* Statistical test: compare food frequency ‘before pandemic’ and ‘during pandemic’ (Indonesia) and ‘before pandemic’ and ‘during COVID-19 lockdown’ (Malaysia) with a Wilcoxon signed rank test.

† Statistical test: compare change in food consumed in rural *v*. change in food consumed in urban using generalised estimating equations (ordinal probit).

### Impact of the pandemic on physical activity

Indonesia, Thailand and Vietnam all showed a self-reported significant decrease in outdoor activities and a significant increase in indoor activities in both rural and urban regions during pandemic (Indonesia) or lockdown (Thailand and Vietnam) (Table 4a). Malaysia showed for urban children older than 7 years a significant increase in moderate-to-vigorous physical activity (16·9 % to 23·3 %). Younger children (3–6 years) showed a significant decrease in physical activity in both rural (78·6 % to 57·1 %) and urban (63·8 % to 53·4 %) areas (Table 4b). For all countries, the use of electronic devices increased (Table 4a and Table 4b).

**Table 4a:** Change in physical activity of SEANUTS II children during the pandemic (Indonesia)/lockdown (Thailand and Vietnam) compared to before COVID-19 pandemic (Indonesia)/before lockdown (Thailand and Vietnam)

	Indonesia				Thailand				Vietnam			
	Rural (*n* 754)		Urban (*n* 744)		Rural (*n* 2063)		Urban (*n* 938)		Rural (*n* 2787)		Urban (*n* 1214)	
	*n*	%	*n*	%	*n*	%	*n*	%	*n*	%	*n*	%
Physical activity outdoor during pandemic/LD												
Decrease (%)	245	32·5	304	40·9	656	31·8	316	33·7	691	24·8	362	29·8
Increase (%)	149	19·8	97	13·0	65	3·2	26	2·8	143	5·1	52	4·3
Unchanged (%)	360	47·7	343	46·1	1227	59·5	514	54·8	1953	70·1	800	65·9
No activity (%)	0		0		115	5·6	82	8·7	0		0	
*P*-value	<0·001		<0·001		<0·001		<0·001		<0·001		**<**0·001	
Indoor activity during pandemic/LD												
Decrease (%)	51	6·8	53	7·1	37	1·8	22	2·3	80	2·9	34	2·8
Increase (%)	236	31·3	284	38·2	494	23·9	239	25·5	759	27·2	387	31·9
Unchanged (%)	467	61·9	407	54·7	1443	69·9	617	65·8	1948	69·9	793	65·3
No activity (%)	0		0		89	4·3	60	6·4	0		0	
*P*-value	<0·001		<0·001		<0·001		<0·001		<0·001		**<**0·001	
Use of electronic devices during pandemic/LD												
Decrease (%)	60	8·0	71	9·5	42	2·0	12	1·3	NA		NA	
Increase (%)	195	25·9	281	37·8	478	23·2	237	25·3	NA		NA	
Unchanged (%)	499	66·2	392	52·7	1424	69·0	628	67·0	NA		NA	
No activity (%)	0		0		119	5·8	61	6·5	NA		NA	
*P*-value	**<**0·001		**<**0·001		**<**0·001		**<**0·001					

LD: LD: lockdown. NA; not available.

Data are reported *n* (%).

Statistical test: %increase = %decrease using a binominal test of ‘before pandemic (ID)/before lockdown (TH and VN)’ and ‘during pandemic (ID)/during lockdown (TH and VN)’ strata.

**Table 4b:** The effect of COVID-19 lockdown on physical activity of SEANUTS II children in Malaysia

	Rural					Urban					
	Before pandemic		During lockdown			Before pandemic		During lockdown			
	*n*	%	*n*	%	*P* ^*^	*n*	%	*n*	%	*P* ^*^	*P* ^†^
Physically active^‡^ (%) (≥ 1 h per day in 3- to 6-year-olds)	22	78·6	16	57·1	0·04	74	63·8	62	53·4	**<**0·001	0·379
Physically active^§^ (%) (≥ 1 h per day of physical activity in 7-year-olds and above	18	17·0	17	16·0	1·00	16	16·9	54	23·3	**<**0·001	0·01
	Mean	sd	Mean	sd		Mean	sd	Mean	sd		
Use of electronic devices for recreation					**<**0·001					**<**0·001	0·588
1 h or less	48	45·3	14	13·2		115	49·4	46	19·7		
2–3 h	55	51·9	90	84·9		112	48·1	183	78·5		
4 h or more	3	2·8	2	1·9		6	2·6	4	1·7		
Use of electronic devices for educational purposes (>7-year-olds)											
1 h or less			38	35·8				74	30·8		
2–3 h			49	46·2				118	80·0		
4 h or more			19	17·9				48	19·4		

Data are reported as mean (s d) or *n* (%).

* Statistical test: compare ‘before pandemic’ and ‘during COVID-19 lockdown’ for ordinal variables using a McNemar rank test.

† Statistical test: compare change in physical activity/use of electronic devices for recreation in rural areas *v*. change in physical activity/use of electronic devices for recreation in urban areas using generalised estimating equations (ordinal probit) analysis.

‡ Physically active was defined as at least 60 min of moderate-to-vigorous physical activity for children 3–6 years of age.

§ For children 7 years or older, physically active was defined as, at least, 60 min per day of moderate-to-vigorous physical activity.

### Impact of the pandemic on food security

Food insecurity increased during the pandemic in Indonesia mainly driven by an increase in individual insecurity and child hunger (both urban and rural). For Malaysia, the lockdown had no significant effect on food insecurity (Table 5).

**Table 5:** The impact of the COVID-19 pandemic on food insecurity in Indonesia and Malaysia (repeated FIQ questionnaire)

	Indonesia											Malaysia										
	Rural (*n* 754)					Urban (*n* 744)						Rural (*n* 188)					Urban (*n* 506)					
	Before pandemic		During pandemic			Before pandemic		During pandemic				Before pandemic		During lockdown			Before pandemic		During lockdown			
	*n*	%	*n*	%	*P* ^*^	*n*	%	*n*	%	*P* ^*^	*P* ^†^	*n*	%	*n*	%	*P* ^*^	*n*	%	*n*	%	*P* ^*^	*P* ^†^
Food secure	200	26·5	160	21·2	0·001	231	31·0	191	25·7	0·001	0·886	104	55·3	109	58·0	0·261	302	59·7	303	59·9	0·211	0·206
Household insecurity	186	24·7	179	23·7		219	29·4	223	30·0			36	19·1	37	19·7		95	18·8	76	15·0		
Individual insecurity	205	27·2	237	31·4		192	25·8	198	26·6			11	5·9	12	6·4		31	6·1	35	6·9		
Child hunger	163	21·6	178	23·6		102	13·7	132	17·7			37	19·7	30	16·0		78	15·4	92	18·2		

Data are reported as *n* (%).

* Statistical tests: Compare % food insecurity ‘before pandemic’ and ‘during pandemic’ (Indonesia) or ‘before pandemic’ and ‘during COVID-19 lockdown’ (Malaysia) using a Wilcoxon signed rank test.

† Statistical tests: Compare the effect of the COVID-19 pandemic on food insecurity in rural and urban areas using a generalised estimating equations (ordinal probit) test.

More than half of the children in Thailand and Vietnam missed their school meals and school milk during COVID-19 lockdown (data not shown).

## Discussion

COVID-19 was declared a pandemic on the 11th of March 2020. With the impeding COVID-19 pandemic, many countries went into partial or full lockdown, all at different timepoints, including the SEANUTS II countries (Table 6). At the time of the outbreak, in Malaysia, school meals were already provided to children from poor households for a long time. This continued when the pandemic started but stopped once schools closed for lockdowns. The programmes were restarted once lockdowns were lifted. In addition to this, monetary assistance was given to the heads of poor households^(16)^. Indonesia did not make any specific food assistance school programmes available to children during the pandemic. There were education programmes developed by selected schools for parents focussing on the importance of providing nutritious food to their children during the pandemic. Children were requested to report their breakfast and lunch meals (e.g. photos of the foods) to their teachers. In Thailand, school lunch programmes and school milk programmes were supplied during the pandemic. All children in child development centres (aged 2–3 years), kindergarten (aged 4–6 years) and primary school (aged 7–12 years) received free lunches and free milk (200 ml per day). Schools in Thailand were generally open during the school year 2020–2021, except for June 2021. As a result of the pandemic, the number of pupils who received nutrition via school feeding programmes decreased. In case schools were closed, meals were not provided at school, but the student’s families were provided with monetary support or vouchers to purchase food. During lockdowns, parent received milk from school for their children^(17)^. The Vietnamese government also provided food assistance programmes to needy families during the pandemic but put no specific school feeding programmes in place. There were also no school meals provided to children during lockdowns and no meals were delivered at home in case of home-based schooling^(18)^. Despite the above-described support efforts by the various countries, the pandemic has led to, income instability, school closures and increased stress levels in parents and guardians that could have compromised their ability to take care of their children’s lifestyle, diet and physical activity. To further analyse this, a specific COVID-19 questionnaire was developed and administered to parents/guardians and their children who participated in the SEANUTS II study, a nationally representative multi-centre survey that was conducted in Malaysia, Indonesia, Thailand and Vietnam between 2019 and 2021. Malaysia administered their COVID-19 questionnaire from June 2020 until August 2020, during COVID lockdown. Schools were closed during this period. In Indonesia, Thailand and Vietnam, children were already going back to school when data collection was conducted during the pandemic. For Malaysia and Indonesia, the COVID-19 analysis can be considered a sub-study of the baseline (main) study because data collection for the baseline (main) study was already terminated because of the outbreak of the pandemic, strict lockdown measures and the high risk of spreading disease. Thailand and Vietnam conducted the COVID-19 analysis alongside the SEANUTS II main study. We cannot exclude that these difference in timing may have affected some analyses results. Certain physical activity behaviours may only have been identified in the data set from Malaysia as strict mobility restriction was in place there during data collection. Changes in these behaviours may have been missed in the other countries. Furthermore, as no data were collected prior to the outbreak of the pandemic, it was not possible for Thailand and Vietnam, in contrast to Malaysia and Indonesia, to make a direct comparison of measurements before and during the pandemic/COVID-19 lockdown. In these countries, the situation before the pandemic could only be assessed by questions from the COVID-19 questionnaire about changes in lifestyle behaviours that were answered from memory (e.g. self-reported). It should be noted that recalling from memory, during the pandemic, lifestyle behaviours from before the outbreak of the pandemic may have yielded biased results. As Indonesia and Malaysia had completed their main study data collection before outbreak of the pandemic they did not solely depend on these self-reported questions from the COVID-19 questionnaire but could also repeat a selection of questions from the main study questionnaires CFH^(4,9)^, FIQ^(10,11)^ and PAQ^(12,13)^. On top of this, Malaysia also repeated some questions from the SES questionnaire about monthly household income and monthly household income spent on food. The repeated measurements yield more accurate/less biased data than those obtained by self-reporting. A strong asset of our COVID-19 analysis is the four-country set-up where almost identical protocols were implemented thereby increasing the generalisability of findings across the countries. Baseline measurements of the proportions of children from rural and urban areas confirmed that de COVID-19 study cohort is representative of the populations of the respective countries as the proportions are very similar to the reported population distributions over these areas^(19)^.

**Table 6:** COVID-19 restrictions in Indonesia, Malaysia, Thailand and Vietnam

Country	Start lockdown	End lockdown	Measures taken
Indonesia	April 2nd 2020		Large-scale social restriction (LSSR) in Jakarta and nearby cities: Depok, Bekasi and Tangerang.
	April 7th 2020		All provincial governments to start taking steps to implement LSSR.
	April 10th 2020	April 23rd 2020	The first LSSR was implemented in all areas of Jakarta and in partial areas of West Java and Banten. Schools, offices, religious activities, public transportation, and other public spaces were temporarily restricted during the LSSR.
	April 24th 2020	June 4th 2020	LSSR, which was initially planned to end on 23 April, was extended to 04 June 2020.
	June 5th 2020	September 10th 2020	This was a transition phase from LSSR to a ‘new normal’. Strict COVID-19 protocols had to be applied, including wearing face masks, physical distancing and a maximum capacity of 50 % for offices, places of worship, recreational facilities, public/mass transportation and conventional and online taxis.
	September 14th 2020	October 11th 2020	The government decided to return to strict LSSR (as before the ‘new normal’) after considering three points: mortality rate, bed occupancy rates at isolation facilities and bed occupancy rates in ICUs of hospitals.
	October 12th 2020	January 11th 2021	Between 6 and 11 October 2020, there was a decrease in daily positive COVID-19 cases. Jakarta’s government re-implemented a transition phase (for the second time).
	January 11th 2021	January 25th 2021	Most areas in Java and Bali islands implemented community activities restrictions enforcement (CARE).
	January 26th 2021	February 8th 2021	It was mandatory for all regions to implement CARE with the following rules: (1) companies/offices should implement work-from-home policy for 75 % of employees. (2) essential sectors in energy, communication, finance and banking could operate with a 100 % capacity with strict COVID-19 protocols. (3) educational activities were still conducted online. (4) dine-in was allowed with a maximum capacity of 25 %. (5) shopping malls and trade centres could operate until 19.00. (6) the maximum capacity of places of worship was 50 %; and (7) restrictions for other activities between 19.00 and 05.00.
	February 9th 2021	June 28th 2021	The government implemented micro-scale activity restrictions. A 50 % maximum capacity of offices, restaurants, and places of worship was still applied, and shopping malls/trade centres could operate until 21.00. Essential sectors had a 100 % of operational hours and capacity with strict COVID-19 protocols.
	July 3rd 2021	July 25th 2021	A surge of COVID-19 cases led the president to declare that emergency CARE should be implemented in Java and Bali islands.
	July 26th 2021	August 2nd 2021	The president decided to extend CARE levels 3 and 4 until 02 August 2021; restrictions of level 3 were more relaxed than level 4. CARE level 3 was for regions with 50–150 COVID-19 cases, 10–30 hospitalised COVID-19 cases and 2–5 COVID-19 mortalities per 100 000 people.
	August 3rd 2021	Now	All restrictions have been lifted.
Malaysia	March 18th 2020	May 3rd 2020	The government declared and enforced the Movement Control Order (MCO): (1) interstate travel not allowed, (2) imposed kindergartens, schools, universities and institutional closures, (3) all religious, social and sports mass gatherings cancelled, (4) all people, including foreign residents were asked to wear face masks (not mandatory), to keep a social distancing of 1 metre and adhere to hand hygiene protocols, and (5) all shops and premises are closed except the essential needs sector and essential activities.
	May 4th 2020	June 9th 2020	The Conditional Movement Control Order (RMCO) was issued by the government: (1) most economic sectors and activities suffered restrictions, (2) sports activities involving large gatherings, body contact or other sports-related factors that increase infection risk are not allowed, and (3) interstate travel is not allowed except for work purposes and to return home after being stranded in hometowns or elsewhere.
	June 10th 2020	October 13th 2020	The government issued the Recovery Movement Control Order (RMCO): (1) economic, educational, religious, hospitality & touristic sector were reopened but with strict standard operating procedures (SOPs). These included meetings, conventions, exhibitions and weddings, (2) the international borders remained closed except for officially approved travelling, (3) pre-schooler and kindergartens resumed operations from July first, 2020, onward, (4) schools reopened in stages (different standards) starting July 15th, wearing face masks mandatory in public spaces from August first, 2020, with violators facing a RM 1000 fine.
	October 13th 2020	December 31st 2020	RMCO and CMCO in different states enforced depending on the local COVID-19 situation.
	January 13th 2021	May 31st 2021	Each state switches between MCO, CMCO and RMCO depending on the local COVID-19 conditions.
	June 1st 2021	June 28th 2021	MCO enforced again, total lockdown.
	June 15th 2021	Now	National Recovery Plan (NRP) is in effect: (1) economic sectors resume operations in stages, (2) starting from October 15th, 2021, schools are allowed to reopen (by states), and (3) by 31st of December 2021, stage 4 NRP allows all gatherings and all economic sectors to reopen. Furthermore, interstate travel according to SOPs and social activities in accordance with SOPs are allowed.
Thailand	March 26th 2020	April 30th 2020	Government declared and enforced a State of Emergency Decree: (1) inter-provincial travel ban, (2) curfew between 22.00 and 04.00, (3) 14-day mandatory quarantine for international travellers, (4) National holidays cancelled (Songkran festival) to prevent massive social gatherings and domestic travel, (5) imposed school closures and restrictions of access to all public spaces except if essential, (6) all international flights were suspended form 4th of April 2020, only emergency or authorised flights were permitted, and (7) all people, including foreign residents, were asked to wear face masks (not mandatory), to keep 2 metres social distance and to adhere to hand hygiene protocols.
	May 1st 2020	Now	Easing of lockdown measures but still under State of Emergency Decree. Various control protocols are still in place.
Vietnam	April 1st 2020	April 26th 2021	In this period, the second and third waves of infections occurred: (1) many provinces/cities were locked down or extended the duration of lockdown according to Directive 16/ CT-TTg. Most facilities were closed. Rotational work assignments for employees were developed to contain the risk of infection, (2) workers had to have travel paper approved by the government organisation or company’s director, (3) curfew between 18pm-5am for many provinces/cities, (4) 14-day mandatory quarantine for international experts (only experts could visit Vietnam, no other travellers were allowed, (5) National holidays were cancelled to prevent massive social gatherings and domestic travel, (6) school closures were imposed, and restrictions were put in place for access to all public spaces except the essential ones. Meetings such as weddings and funerals, etc., were prohibited for gatherings of more than 10 people, and (7) all people including foreign residents were asked to wear face masks (although this was not mandatory), to keep a physical distance of more than 2 metres and to adhere to hand hygiene protocols.
	April 27th 2021	September 30th 2021	In this period, the fourth wave of infections occurred.
	October 1st 2021	Now	Most restrictions have been lifted. Vietnam has moved to controlling the COVID-19 pandemic according to Resolution No. 128/NQ-CP of the Government.

For Indonesia, Thailand and Vietnam, intake of most food groups did not change during pandemic/lockdown compared to before pandemic for most children (60·0–95·0 %), based on self-reporting (COVID-19 questionnaire). For the minority of children that did change their food intake during the pandemic/lockdown, the intake of almost all food groups decreased. Exceptions are an increase in the consumption of rice/cereals (rural areas) and larger portion size of main meals in Indonesia and an increased consumption of eggs, milk, rice/cereals (only in rural regions) and larger portion size of main meals in Thailand. Interestingly, in Vietnam, the self-reported consumption of all food groups decreased during lockdown including the portion size of main meals.

Only in rural Thailand some marginal decreases in food intakes from the period during the lockdown persisted after lockdown (data not shown). Most subjects who self-reported a decrease for a certain food group during lockdown reported no change or an increased intake of the respective food group after lockdown. Likewise, most subjects who self-reported an increase for a certain food group during lockdown reported no change or a decreased intake of the respective food group after lockdown. This may partly be explained by regression to the mean.

For Indonesia, results from the repeated CFH questionnaire were not identical to the results from the self-reported changes in foods consumed in the COVID-19 questionnaire (Table 3a *v*. Table 3b, Indonesia). The repeated CFH measurements showed that vegetable consumption had increased in rural as well as urban Indonesia during the pandemic while a decrease in vegetable consumption was self-reported for children in rural Indonesia via the COVID-19 questionnaire. The exact phrasing of the respective questions can partly explain this discrepancy but also the fact that in the CFH questionnaire parents/guardians were asked to report food intake over the previous week while in the COVID-19 questionnaire parents/guardians were asked to call to memory food intake from a much longer time ago is of major significance. For these reasons, the repeated CFH is more accurate than the COVID-19 questionnaire. Interestingly, for Malaysia, the lockdown resulted in a healthier dietary pattern with more basic food groups and less discretionary foods. The repeated CFH questionnaire showed an increased consumption of vegetables, fruits and eggs but decreased consumption of milk and dairy products. It also showed a decreased intake of sweetened beverages in Malaysian children during lockdown. This might be explained by the fact that there was more time to cook and eat at home during the pandemic, the fact that the Malaysian government recommended the consumption of vegetables and fruits to support the immune system and the disruption of school milk programmes due to school closure. These observations partly replicate the observations made by UNICEF and UNFPA who showed that, on average, Malaysian households consumed more eggs (+50·0 %), rice (+40·0 %) and instant noodles (+40·0 %), and less snacks and sweets (–62·0 %) and fruits (–40·0 %) during lockdown than before the pandemic. Low-income households, who earned below RM2,000 per month (∼$420 USD, conversion date November 2022), spent more on eggs (+5·0 %) and instant noodles (+8·0 %) relative to higher earning groups and less on protein (32·0 % *v*. 17·0 % in higher-income households) and rice (19·0 % *v*. 7·0 % in higher-income groups) during lockdown^(18)^.

The pandemic not only had nutritional consequences but also negatively impacted socioeconomic and food security parameters. In all countries, monthly household income decreased as many people lost their jobs. Food security in Indonesia decreased as well. These socioeconomic effects of the pandemic have also been found in other studies^(20–22)^. Interestingly, only in Malaysia did food expenditure increase during the lockdown period. This was not observed in any of the other countries. It is possible that the financial support in Malaysia led to more money available to be spent on food. Increased household size during lockdown and the use of financial savings for food purchases may further have contributed to the increased food spent in Malaysia. The fact that there were no school meal/milk programmes available during lockdown may also have contributed.

Outdoor physical activity decreased during lockdown while indoor physical activity increased in Indonesia, Thailand and Vietnam. For Vietnam, it had been reported that, because of social distancing and school closures, children had more time for online activities, but less for physical exercise. Moreover, parents less strictly managed their children’s screen time^(18)^. In Malaysia, overall physical activity increased during lockdown for older children with low baseline PAL levels (>7 years) and significantly decreased in younger children (3–6 years). This may be explained by the fact that, during the pandemic, there could have been more leisure time to do physical activity at home for the older children while sedentary screen time for the younger children was more permitted as would have normally been the case by the parents/guardians as they were working from home or busy with household chores. It is noteworthy that physical activity and sedentary screen time seem to have been less impacted by the pandemic in low- and middle-income countries than in high-income countries^(23)^.

Electronic device usage increased in all countries. This can at least be partly explained by the fact that many children were still doing much of their learning through online education^(18,24)^.

In conclusion, the COVID-19 pandemic impacted the lives of SEANUTS II children and their families differently, both negatively as well as positively. Understanding these lifestyle behaviour changes in each country may help public health authorities reshape future policies on nutrition and lifestyle recommendations when new pandemics arrive, and lockdown policies are implemented. Future policies should include nutrition-focused social protection programmes and food assistance programmes for children from impacted households, recommendations to children to be physically active at home and stimulation of parents to engage with their children and stimulate them to play more fun physical activities/games at home. Governments and public health authorities should pay particular attention to those households that are still food secure but on the brink of insecurity as a decrease of monthly household income and loss of jobs are the main drivers of the devastating effects of any pandemic. Physical activity and eating healthy, nutrient-adequate diets should be promoted to increase the overall resilience of the population. Of interest to note in this respect are the more general learnings from the SEANUTS countries, based on their experience, with respect to the COVID-19 pandemic: (1) investment in health facilities is key^(17,25–29)^, (2) universal health coverage needs to be in place to guarantee that all COVID-19 patients will have access to essential treatment without financial barriers^(17,25,26,30)^, (3) the contribution of health volunteers is of crucial importance to control the pandemic^(17,25,26,31)^, (4) action needs to be taken early^(17,25,26,29,32)^ and (5) nationwide public cooperation on effective social measures is required to effectively combat a pandemic^(17,25,33)^. Especially, the affordability of healthy and nutrient-adequate diets remains an important focus area considering the ongoing rising food prices, inability to import foods and decreased production of fruits and vegetables due to farm closures and worker shortages^(34–37)^.
